# Coordinated trafficking of synaptic vesicle and active zone proteins prior to synapse formation

**DOI:** 10.1186/1749-8104-6-24

**Published:** 2011-05-10

**Authors:** Luke AD Bury, Shasta L Sabo

**Affiliations:** 1 Department of Pharmacology, Case Western Reserve University School of Medicine, Cleveland, Ohio, 44106, USA; 2 Department of Neuroscience, Case Western Reserve University School of Medicine, Cleveland, Ohio, 44106, USA

## Abstract

**Background:**

The proteins required for synaptic transmission are rapidly assembled at nascent synapses, but the mechanisms through which these proteins are delivered to developing presynaptic terminals are not understood. Prior to synapse formation, active zone proteins and synaptic vesicle proteins are transported along axons in distinct organelles referred to as piccolo-bassoon transport vesicles (PTVs) and synaptic vesicle protein transport vesicles (STVs), respectively. Although both PTVs and STVs are recruited to the same site in the axon, often within minutes of axo-dendritic contact, it is not known whether or how PTV and STV trafficking is coordinated before synapse formation.

**Results:**

Here, using time-lapse confocal imaging of the dynamics of PTVs and STVs in the same axon, we show that vesicle trafficking is coordinated through at least two mechanisms. First, a significant proportion of STVs and PTVs are transported together before forming a stable terminal. Second, individual PTVs and STVs share pause sites within the axon. Importantly, for both STVs and PTVs, encountering the other type of vesicle increases their propensity to pause. To determine if PTV-STV interactions are important for pausing, PTV density was reduced in axons by expression of a dominant negative construct corresponding to the syntaxin binding domain of syntabulin, which links PTVs with their KIF5B motor. This reduction in PTVs had a minimal effect on STV pausing and movement, suggesting that an interaction between STVs and PTVs is not responsible for enhancing STV pausing.

**Conclusions:**

Our results indicate that trafficking of STVs and PTVs is coordinated even prior to synapse development. This novel coordination of transport and pausing might provide mechanisms through which all of the components of a presynaptic terminal can be rapidly accumulated at sites of synapse formation.

## Background

In the cortex, the bulk of synapse formation occurs at high rates over a period of several weeks during early postnatal development. Formation of individual synapses is triggered when axons and dendrites contact one another [1,2]. For these axo-dendritic contacts to stabilize and form a synapse, synaptic vesicle and active zone proteins must be recruited to the site of contact very rapidly, within minutes to hours [3-10]. This rapid assembly is remarkable, considering that each presynaptic protein needs to be transported from the soma to individual sites of synapse formation along the axon.

Recent work using live imaging of developing neurons has revealed mechanisms that might facilitate rapid synapse assembly. For example, rather than transport each molecule individually, neurons package multiple presynaptic components into vesicles in the cell body and transport the entire group together. Two major groups of proteins are transported in this fashion: active zone proteins are transported in piccolo-bassoon transport vesicles (PTVs), while synaptic vesicle-associated proteins are transported in synaptic vesicle protein transport vesicles (STVs) [4,9,11-17]. PTVs and STVs can be distinguished both morphologically and biochemically. PTVs have been described as dense-core vesicles or aggregates of vesicles and proteins that range in size from approximately 80 nm in diameter for dense core vesicles to 130 nm by 220 nm in area for aggregates [12,16]. Proteins transported by PTVs include piccolo, bassoon, N-cadherin and syntaxin [11,12,16,18,19]. STVs are heterogeneous in size and shape and are composed of both tubulovesicular and clear core vesicles [9,13,14]. Proteins carried by STVs include synaptophysin, synapsin Ia, synaptotagmin and synaptobrevin/vesicle-associated membrane protein 2 (VAMP2) [4,9,13,14,16]. Both types of vesicles are packaged via the trans-Golgi network before being transported through the axon [14,16,20].

Rapid assembly of presynaptic terminals can also be facilitated by having the source of synaptic proteins nearby and readily available. Indeed, many STVs and PTVs are mobile within axons well before synapse formation. STVs and PTVs travel in a saltatory fashion in both the anterograde and retrograde directions along the entire length of the axon [4,8,11,13-15,17,19]. This provides a readily available pool of synaptic vesicle and active zone proteins wherever and whenever axo-dendritic contact occurs.

Since both STVs and PTVs must be delivered to the same site during synapse assembly, we hypothesized that trafficking of STVs and PTVs might be coordinated even prior to synapse assembly, providing an additional mechanism to facilitate rapid accumulation of the full complement of proteins required for synaptic transmission. Such coordination could arise through co-transport of STVs and PTVs, through pausing of STVs and PTVs at the same sites, or both. Coordinated pausing presents a particularly intriguing possibility since sites along the axon where STVs pause their transport are preferential sites of synapse formation [4]. It was previously suggested that cues that control pausing at these sites could promote rapid synapse assembly by increasing the probability that STVs are at or near any given site when axo-dendritic contact occurs. If this model is correct, then synapse assembly would be most efficient if PTVs are also attracted to these same sites. However, it is not yet known whether PTVs also pause at these sites and, if so, whether they do so simultaneous with STVs. A recent report demonstrated that PTVs and STVs (particularly those resembling small, clear vesicles) can be observed tethered together in electron micrographs of developing axons [16]. These aggregates could correspond to PTVs and STVs that are either being co-transported or pausing at the same site at the same time.

Here, we tested whether transport and pausing of PTVs and STVs is coordinated prior to synapse assembly using time-lapse confocal imaging of green and red fluorescent protein-tagged synaptic vesicle and active zone proteins within the same axon. We found that a significant portion of STVs and PTVs move together and share pause sites. Interestingly, both STVs and PTVs preferentially paused at these sites when another vesicle was present. These observations raised the question of whether a direct interaction between STVs and PTVs coordinates their transport and pausing. Reducing PTVs in the axon minimally affected the movement and pausing of STVs, arguing against this mechanism and suggesting that other unidentified signals are responsible for STV and PTV coordination. These findings represent novel mechanisms that can facilitate the rapid recruitment of presynaptic proteins to the same site within the axon and, therefore, promote synapse development.

## Results

### STVs and PTVs can move together

STVs and PTVs move in a similar saltatory fashion within the axon in both the anterograde and retrograde directions [4,9,11,13,14,19,21]. However, because STVs and PTVs have almost always been imaged separately, it is not yet known if and how STV and PTV trafficking interrelate. To determine this, we used time-lapse imaging of neurons co-transfected with fluorescent STV and PTV markers. In this assay, 5- to 6-days *in vitro *(DIV) rat cortical neurons were co-transfected with GFP-bassoon to label PTVs and either synaptophysin-mcherry or synaptophysin-monomeric red fluorescent protein (mRFP) to label STVs. Previous studies have indicated that fluorescent proteins attached to bassoon and synaptophysin effectively label PTVs and STVs, respectively [8,9,11,14,15,17,20,22]. One to two days after transfection, time-lapse images of the fluorescently labelled vesicles were obtained from individual axons. Images were taken every 10 s for 7.5 minutes. An example is shown in Movie 1 in Additional file 1.

**Figure 1:**
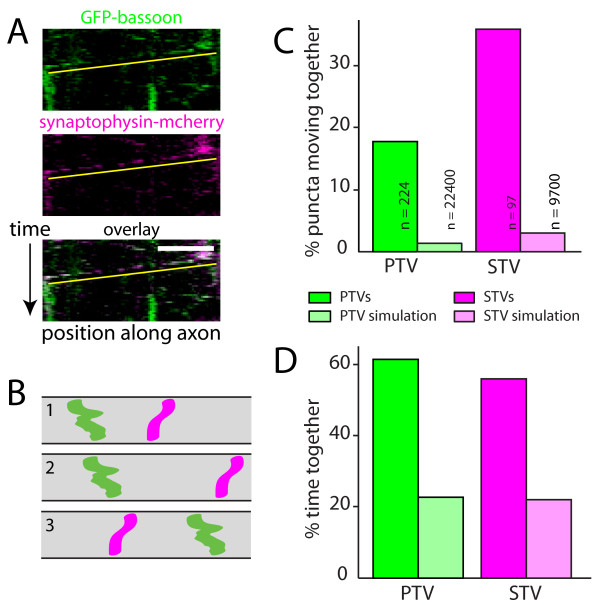
**STVs and PTVs move together**. **(A) **Kymographs of an axon segment showing the movements of PTVs (GFP-bassoon; green) and STVs (synaptophysin-mcherry; magenta) in the same axon. Yellow lines underline the moving STVs and PTVs. Bottom panel: overlay of STV and PTV fluorescence. On the ordinate axis, one pixel corresponds to 10 s. On the abscissa, the scale bar corresponds to 10 μm. **(B) **Simulations of model axons were performed by randomizing initial positions of vesicles while maintaining movement and pausing characteristics of the original imaged vesicles. Diagrams illustrate kymographs of three simulations of model axons. Movements of two vesicles are shown in each model axon. **(C) **Plot showing the percentage of PTVs that move with STVs (green) and STVs that move with PTVs (magenta). PTV and STV simulations (light green and light magenta, respectively) correspond to the predicted values from the simulations. **(D) **Quantification of the percentage of time that PTVs and STVs spent together. Both the percentage of vesicles that moved together and the time spent together were substantially larger in the data than is predicted by chance (simulations).

From Additional file 1 and the kymographs in Figure 1A, it is clear that PTVs and STVs moved together in both anterograde and retrograde directions. To quantify these correlated movements, the positions of individual STV and PTV puncta were tracked independently while blind to the other channel, and then their movements were compared. To determine whether these correlated movements could be accounted for by chance, the experimental data were ultimately compared to model axons where the initial positions of all of the puncta were randomized but the density of puncta and properties of their movements were derived from the experimental data (Figure 1B).

During the imaging period, 18.3% of PTVs moved with STVs (n = 224 vesicles), while 37.1% of STVs moved with PTVs (n = 97 vesicles; Figure 1C). STVs and PTVs that moved together also spent the majority of their time together (Figure 1D). The prevalence of correlated STV and PTV movements was over ten-fold higher for the experimental data than predicted by the same analysis of model axons (Figure 1C; model, n = 22,400 PTVs and 9,700 STVs). In addition, the percentage of time STVs and PTVs spent moving together was over 2.5-fold higher than expected by chance (Figure 1D). These data indicate that populations of STVs and PTVs travel together within the axon, which could allow both sets of presynaptic proteins to be distributed together to sites of synapse formation.

### STVs and PTVs display similar pausing characteristics

**Figure 2:**
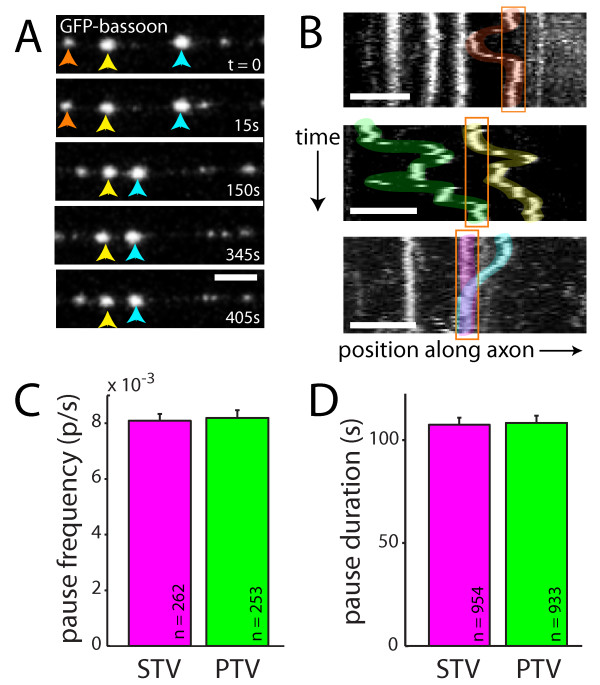
**PTV pausing is qualitatively and quantitatively similar to STV pausing**. **(A) **Time-lapse images of PTVs labeled with GFP-bassoon. Individual PTVs are tracked with red, yellow or blue arrows. Most PTVs paused, and pauses were of varied durations. Frames were collected at the indicated times. Scale bars: 5 μm. **(B) **Kymographs demonstrating the movements of PTVs in three axons. Individual PTVs paused repeatedly at the same site (top panel), and multiple PTVs paused at the same site (middle and bottom panels). Pausing of multiple PTVs at a given site occurred both sequentially (middle panel) and simultaneously (bottom panel). Individual vesicles are highlighted in different colors for visualization. Pause sites are outlined in orange. On the ordinate axis, one pixel corresponds to 10 s. **(C,D) **STVs (magenta) and PTVs (green) paused at similar frequencies (C) and for similar mean durations (D). Data represent the mean + standard error.

Although the movements of both STVs and PTVs have been extensively described individually, the pauses between movements have been less thoroughly analyzed and have not been compared. Comparing STV and PTV pausing behavior is important since STVs preferentially pause at sites of eventual synapse formation [4,9]. When PTV behavior was examined, it was clear that PTV pausing exhibited many of the properties previously described for STVs [4] (Figure 2A,B). For example, the same PTV could be observed repeatedly pausing at a given site (Figure 2B, top panel). In addition, multiple PTVs paused at the same site, both sequentially and simultaneously (Figure 2B, middle and bottom panels, respectively). When STV movements were quantified, the mean STV pause frequency was 0.0081 ± 0.0002 pauses/s (n = 262 vesicles; Figure 2C). The average STV pause duration was 107.4 ± 3.4 s (n = 954 pauses; Figure 2D). Both measurements concur with previous observations [4]. As shown in Figure 2C,D, PTV pause frequency and duration in 7- to 8-DIV neurons were nearly identical to those in the STV data, with PTVs pausing at a frequency of 0.0082 ± 0.0003 pauses/s and average duration of 108.3 ± 3.5 s (n = 253 vesicles, n = 933 pauses). The similarities between the pausing behavior of STVs and PTVs raise the question of whether PTVs might pause with STVs at sites of eventual synapse formation. Like STVs [4], regulation of PTV pausing could be an important target of signals that control synapse assembly.

### STVs and PTVs pause simultaneously at the same sites in the axon

**Figure 3:**
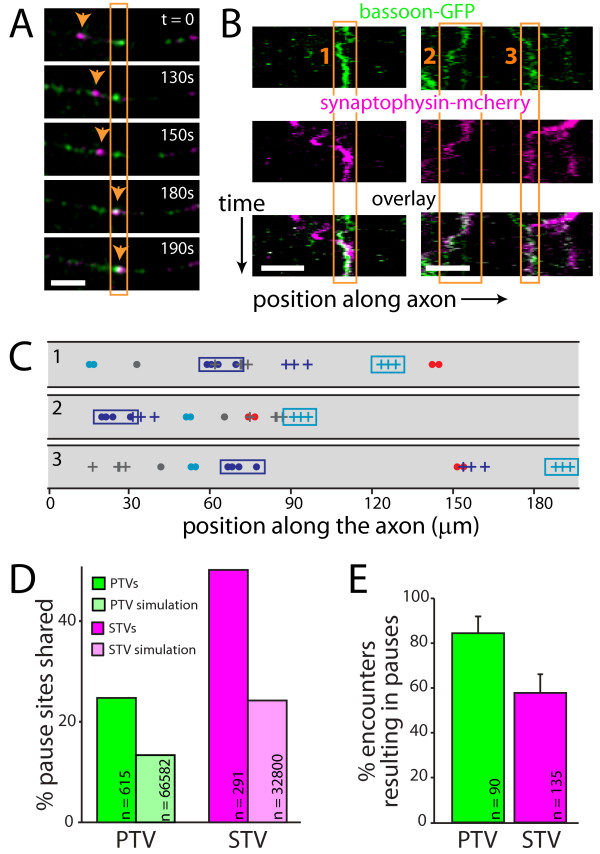
**STVs and PTVs pause at the same sites**. **(A) **Time-lapse images of a segment of axon expressing both synaptophysin-mcherry (magenta) and GFP-bassoon (green). In each panel, fluorescence signals from the two channels are overlaid, and co-localization is indicated by white. The orange box outlines a site where a PTV is paused. The arrow tracks a STV that then pauses at that site. Scale bars: 5 μm. **(B) **Kymographs showing two examples of STVs and PTVs that pause at the same sites. On the ordinate axis, one pixel corresponds to 10 s. Bottom panel: overlay of STV and PTV fluorescence. Orange boxes outline three shared pause sites. **(C) **Simulations of model axons were performed by randomizing initial positions of vesicles while maintaining movement and pausing characteristics observed for the original experimental vesicles. Diagrams show the superimposed locations of all pause sites for individual STVs and PTVs in model axons. Three simulations of the same experimental data are shown. STV pause sites are indicated with a dot while PTV pause sites are indicated with a plus sign. Each color represents an individual vesicle. Cyan and magenta boxes outline the pause sites of the same PTV and STV, respectively, in each model axon. For each axon imaged, 100 such simulations were performed, allowing estimation of the degree of co-transport and co-pausing expected from chance alone. **(D) **Plot illustrating the percentage of PTV (green) and STV (magenta) pause sites that are shared with STVs and PTVs, respectively. The fraction of shared sites is much higher than predicted by chance (via simulations, light green and light magenta). **(E) **A large majority of PTVs that encountered STV pause sites paused at those sites. Similarly, most STVs that encountered PTV pause sites then paused at those sites. Error bars display the 95% confidence interval.

For a synapse to develop, both active zone and synaptic vesicle proteins need to be recruited to the same site. Sharing a pause site provides a potential mechanism for recruiting both STVs and PTVs to the same spot in the axon. Since places where STVs pause are preferred sites of synapse formation, it seemed likely that PTVs also pause at these sites. To test this hypothesis, STVs and PTVs were imaged and analyzed as described above. From the time-lapse panels and kymographs shown in Figure 3A,B, it is clear that both types of vesicles paused at sites where the other type of vesicle also paused.

**Figure 4:**
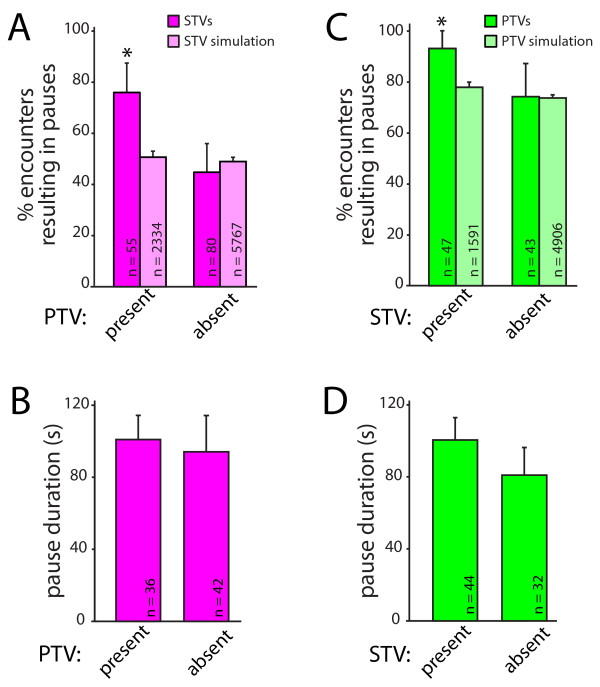
**STVs and PTVs preferentially pause at the same sites at the same time**. **(A) **STVs (magenta) are significantly more likely to stop at a PTV pause site when PTVs are at the site. This preference cannot be accounted for by chance since no dependence on the presence of PTVs was seen in simulations (light magenta). The data presented correspond to the mean + 95% confidence intervals; *95% confidence intervals do not overlap. **(B) **STVs pause for similar lengths of time regardless of whether a PTV is present at the pause site. Data are presented as mean + standard error. **(C) **PTVs (green) are more likely to pause at a site when an STV is present. The same dependence was not observed in simulations (light green). **(D) **PTVs pause for similar lengths of time, regardless of whether STVs are present at the pause sites. Data are the mean + standard error.

To determine whether pausing of STVs and PTVs at the same sites was significantly greater than would be predicted by chance, pausing at the same sites was quantified in each axon then compared to simulations in model axons (Figure 3C). For STVs, 47.9% of pauses were at sites where a PTV also paused (n = 305 pauses; Figure 3D), compared to 24.5% using the same analysis of model axons (n = 33,967 pauses; Figure 3D). This indicates that STVs preferentially pause at PTV pause sites. Likewise, 24.5% of all PTV pauses were at sites where an STV also paused (n = 620 pauses; Figure 3D), compared to 14.0% using the same analysis of model axons (n = 69,263 pauses; Figure 3D), indicating that PTVs preferentially pause at STV pause sites. Some STVs do not encounter PTV sites during our imaging time window and *vice versa*. To account for this, we also quantified pausing at the same site with the analysis limited to STVs and PTVs that had the opportunity to pause at PTV and STV sites, respectively (Figure 3E); 57.8% of STVs that encountered a PTV site paused (n = 135), and 84.4% of PTVs that encountered STV sites paused at those sites (n = 90). PTVs are more likely to pause at STV sites than *vice versa*; however, this difference appears to be an inherent property of the vesicles since it is also observed in model axons (Figure 4A,C). It has been shown previously that STV pause sites are preferred sites of synapse formation [4], and PTVs pause at these same sites; therefore, PTVs pause at sites of synapse formation.

Since both PTVs and STVs pause in the same places, the next question addressed was whether STVs and PTVs stop at these sites at the same time. Synapse assembly is a rapid process, and concurrent attraction of both STVs and PTVs could provide a mechanism for efficient assembly of presynaptic terminals. To determine whether PTVs and STVs are simultaneously attracted to sites of synapse formation, we quantified whether STVs preferentially pause at PTV pause sites when a PTV is present. As shown in Figure 4, STVs were more likely to pause at a site that contained a PTV than a site that did not. When STVs encountered pause sites with PTVs present, 76.4 ± 11.2% of STVs paused with the PTV (n = 55; Figure 4A). In contrast, only 45.0 ± 10.9% of STVs paused at PTV pause sites when a PTV was not there (n = 80). This 1.7-fold difference in the likelihood of pausing with a PTV could not be accounted for by chance because there was no interaction between STVs and PTVs when the same analysis was performed on data obtained from simulations with model axons (Figure 4A). In the model, 52.4 ± 1.9% of STVs paused at PTV pause sites with a PTV present (n = 2,334), and 51.6 ± 1.3% of STVs paused at PTV pause sites without PTVs present (n = 5,767). The pause duration of an STV stopped at a PTV pause site was also measured. In contrast to the likelihood of pausing, the pause duration of STVs is not affected by the presence or absence of PTVs (Figure 4B). The average pause duration for STVs paused at sites with PTVs present was 101.0 ± 13.4 seconds (n = 42 pauses), while the average pause duration for an STV paused at a site without a PTV present was 94.2 ± 20.1 seconds (n = 36).

It is not yet known whether STVs and PTVs must be recruited to nascent presynaptic terminals in a defined order, so we also tested whether PTVs are more likely to pause at sites that contain STVs. As shown in Figure 4C, PTVs paused at sites that contained STVs at a significantly higher rate (93.6 ± 7.0%, n = 47) than at sites where STVs had previously paused but were no longer present (74.4 ± 13.0%, n = 43). In contrast, this effect of STVs on PTV pausing behavior was not seen with analysis of the randomized model: in the simulations, 76.0 ± 2.0% of PTVs paused at STV sites with an STV present (n = 1,591), and 74.7 ± 1.1% of PTVs paused at STV sites without STVs present (n = 4,906). There was an equal probability that either vesicle arrived first at a shared pause site (based on 95% confidence interval; data not shown). Similar to STVs pausing at PTV sites, the mean pause duration of PTVs pausing at STV pause sites is not affected by the presence of STVs (Figure 4D). The average pause duration for PTVs paused at sites with STVs present was 100.5 ± 12.4 s (n = 44 pauses), while the average pause duration for PTVs paused at sites without STVs present was 80.9 ± 15.3 s (n = 32 pauses). These data demonstrate that PTVs prefer to pause at sites that contain STVs and *vice versa*, suggesting that STVs and PTVs are attracted to the same sites at the same time.

### Recruitment of a PTV enhances accumulation of additional PTVs at sites of synapse formation

**Figure 5:**
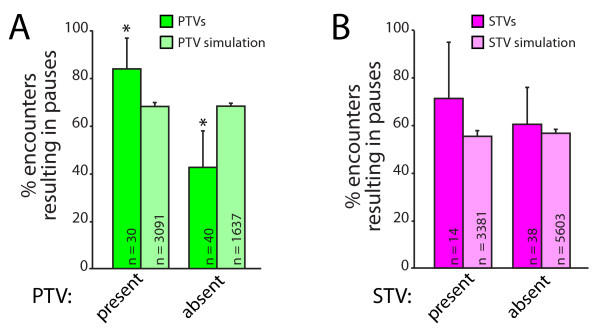
**Multiple PTVs are attracted to the same sites**. **(A) **PTVs are more likely to pause when other PTVs are present (green). Spatially randomized simulations are shown in light green and cannot account for the tendency of PTVs to pause simultaneously with other PTVs. **(B) **STVs are not more likely to pause when other STVs are already present (magenta). Simulations are shown for comparison (light magenta). Error bars represent 95% confidence intervals; *95% confidence intervals do not overlap.

Assembly of presynaptic terminals involves recruitment of multiple PTVs [11]. Therefore, we wondered whether PTVs might also increase attraction of other PTVs to sites of synapse formation. To test this, we quantified the percentage of PTVs that paused at sites where another PTV was either present or had previously paused. When a PTV was already anchored at a particular pause site, there was a high likelihood of an additional PTV pausing at that site: 83.3 ± 13.3% of PTVs paused when they encountered sites with other PTVs (n = 30; Figure 5A). However, when a PTV was not present at the pause site, the likelihood that a PTV would pause was significantly lower (42.5 ± 15.3%, n = 40). This nearly two-fold increase in attraction of PTVs to sites containing other PTVs could not be explained by chance since the increase was not observed in our simulations. In model axons, PTVs paused at 67.1 ± 1.6% of sites with other PTVs (n = 1,267) and 68.1 ± 1.2% of sites without other PTVs (n = 5,827; Figure 5A). This indicates that the presence of a PTV at a pause site promotes recruitment of additional PTVs to that site.

Interestingly, the presence of an STV at a particular pause site did not significantly increase the probability of an additional STV pausing at that site (chance of pausing with another STV present = 71.4 ± 23.7%, n = 14; without another STV present = 60.5 ± 15.5%, n = 38; Figure 5B). As expected, no dependence of STV pausing on the presence of other STVs was observed in our randomized model axon (STV present = 56.8 ± 2.4%, n = 1,665; STV absent = 59.8 ± 1.6%, n = 3,443; Figure 5B). These data suggest that STVs do not interact with one another in a way that promotes recruitment to sites of synapse formation.

### STV pausing is only mildly dependent on PTVs

**Figure 6:**
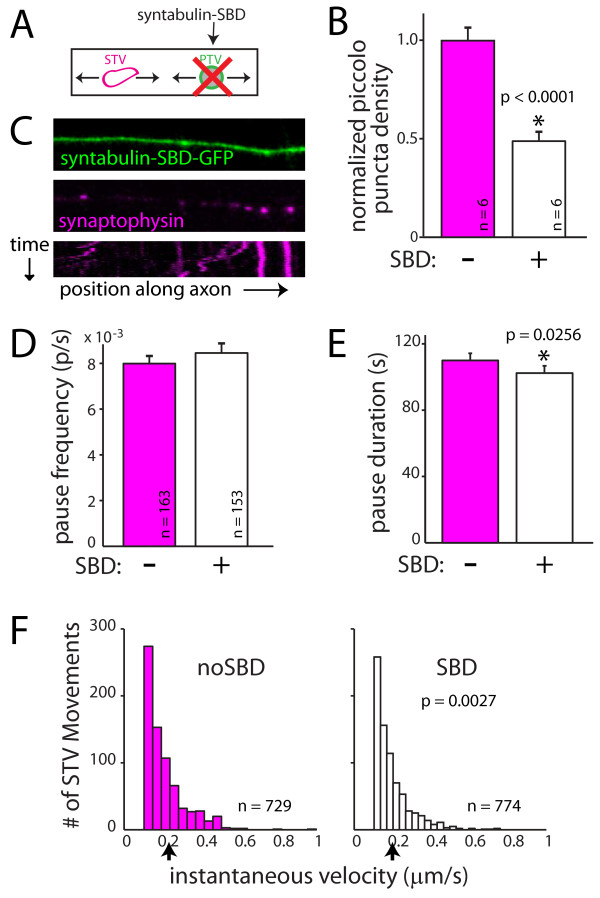
**A direct interaction between STVs and PTVs cannot account for the attraction of STVs to pause sites that contain PTVs**. **(A) **PTV transport was inhibited using dominant-negative syntabulin (syntaxin binding domain, SBD) fused to GFP. **(B) **Expression of syntabulin-SBD-GFP decreases the density of PTV puncta in axons when compared to axons expressing GFP alone. PTVs were identified by immunofluorescent labeling for endogenous piccolo. **(C) **Images and kymograph (bottom) showing that STVs (magenta) move and pause in SBD-expressing axons (green). **(D) **The frequency of STV pausing was unchanged in the presence of SBD-GFP. **(E) **STV pause durations are shorter when PTV localization in the axon is disrupted. The change in pausing upon expression of SBD-GFP is not sufficient to account for the coordinated pausing of STVs and PTVs. In B, D and E, error bars correspond to the standard error. *, difference is significant. *P*-values are from Wilcoxon rank-sum test and are indicated in the figure. **(F) **The instantaneous velocities of STVs are increased in SBD-expressing axons. Black arrow, mean instantaneous velocity.

The data presented above demonstrate that pausing of STVs at sites of synapse formation is enhanced at sites that contain PTVs and *vice versa*. This raises the question of whether a direct physical interaction between STVs and PTVs is important for recruitment of synaptic proteins to sites of synapse formation. If so, then STV pausing should be altered if PTVs are decreased or eliminated from the axon. Conveniently, a method for disrupting PTV transport - and, therefore, decreasing PTV density in the axon - has recently been described [18,19]. This approach utilizes a dominant negative construct that disrupts the connection between the PTV-associated protein syntaxin and syntabulin, which links PTVs to the kinesin motor KIF5B. This construct mimics the syntaxin binding domain (SBD) of syntabulin and interferes with the syntaxin-syntabulin interaction. Expression of syntabulin-SBD has been shown to specifically prevent the majority of PTVs from being transported out of the cell body and into the axon without directly affecting STV transport or other KIF5 cargo [18,19] (Figure 6A). Imaging of our cortical cultures confirmed that the density of endogenous piccolo puncta is dramatically decreased in axons of neurons expressing syntabulin-SBD fused to GFP when compared to neurons expressing GFP alone (Figure 6B).

Consistent with the published data, transfection of neurons with the syntabulin-SBD construct did not interfere with STV transport in axons (Figure 6C): STVs still moved, and the mean distance moved over the course of imaging was unchanged (no SBD = 8.90 ± 0.93 μm, n = 163 vesicles; SBD = 9.30 ± 0.82 μm, n = 153 vesicles; *P *= 0.68). When STV pausing was quantified, expression of the syntabulin-SBD construct yielded no change in the pause frequency (no SBD = 0.0085 ± 0.0003 pauses/s, n = 163 vesicles; SBD = 0.0080 ± 0.0004 pauses/s, n = 153 vesicles; *P *= 0.62; Figure 6D) but did cause a slight decrease in the average duration of STV pauses (no SBD = 109.9 ± 4.4 s, n = 586 pauses; SBD = 102.4 ± 4.3 s, n = 582 pauses; *P *= 0.03; Figure 6E). In addition, the instantaneous velocities of STVs were slightly reduced in SBD-transfected neurons (no SBD = 0.208 ± 0.004 μm/s, n = 729 movements; SBD = 0.193 ± 0.004 μm/s, n = 774 movements; *P *= 0.003; Figure 6F). Although there was a decrease in STV pause duration when PTV transport was disrupted, the magnitude of the effect and the lack of a change in the probability of pausing argue against PTVs themselves controlling STV pausing. These data suggest that a direct interaction between STVs and PTVs is not responsible for the increased attraction of STVs to sites that contain PTVs.

## Discussion

Synaptic vesicle and active zone proteins assemble rapidly at stable axo-dendritic contacts. Here, we hypothesized that trafficking of STVs and PTVs is coordinated even prior to synapse assembly, which could facilitate rapid assembly. By performing live multi-channel fluorescence confocal imaging in neurons expressing both synaptophysin-mRFP and GFP-bassoon, we were able to record and analyze the movements of STVs and PTVs simultaneously in the same neuron. Our results indicate that STVs and PTVs are coordinated during transport and before stabilization since PTVs and STVs move together within the axon. We also find that STVs and PTVs pause at the same sites within the axon, particularly when the other type of vesicle is also paused at that site. This attraction of STVs and PTVs to the same pause sites is not mediated by a direct interaction between STVs and PTVs since reducing the density of PTVs in the axon yielded only small changes in STV pausing. In summary, our data support a model of synapse formation in which STV and PTV trafficking is coordinated even prior to axo-dendritic adhesion. This coordination includes coincident stopping of STVs and PTVs at predefined sites of synapse formation, independent of a direct interaction between STVs and PTVs.

### Is STV and PTV transport coordinated?

Although it is clear that both PTVs and STVs need to be recruited to the same sites in order for pre-synaptic terminals to develop, it is not immediately apparent as to how they both arrive at the same destinations. Previously, STV and PTV dynamics have been imaged only separately, leaving it unknown whether they are transported together [4,9,11-15,17,21,23]. Recent work showed that clear, synaptic vesicle protein-containing vesicles and dense-core, PTV-like vesicles can be seen apparently tethered together in electron micrographs of young neurons [16]. This was the first indication that PTVs and STVs might be transported together, but it remained unclear whether these vesicle aggregates correspond to either STVs and PTVs being transported together or paused together or a later stage in synapse development. Our analysis indicates that a sizable portion of STVs and PTVs move with each other and spend the majority of their time together. STVs and PTVs often moved separately before or after moving together, suggesting that STVs and PTVs can move with each other while also maintaining their separate identities. This observation indicates that, although it is possible that some STVs contained molecules of GFP-bassoon and/or a portion of PTVs contained molecules of synaptophysin-mRFP, missorting of STV and PTV marker proteins cannot account for the observed co-transport of STVs and PTVs. Coordinating STV and PTV movement could represent a mechanism for rapid synapse development, since a full complement of synaptic proteins could immediately be delivered to a potential synaptic site. It will be important in the future to determine whether co-transport is mediated by a direct interaction between STVs and PTVs or by other mechanisms.

Previously, it was shown that STV pause sites are preferred sites of synapse formation [4], raising the question of whether PTVs also tend to pause at these same sites of synapse formation. By labeling both STVs and PTVs within the same axon, we were also able to compare spatial and temporal properties of pausing for both types of vesicles. STV and PTV pausing were qualitatively and quantitatively similar. Like STVs, multiple PTVs paused at the same sites, and the same PTVs returned repeatedly to a given site. The frequency of pausing and duration of pauses for STVs and PTVs were also similar. Importantly, STVs and PTVs paused at the same sites within the axon. This implies that PTVs also pause at predefined sites of synapse formation. The propensity of STVs and PTVs to preferentially pause at the same sites within the axon suggests that there is a mechanism that recruits both types of vesicles to the same site at the same time. These same mechanisms could be utilized by axons to recruit the necessary proteins during synapse formation.

### What causes STV and PTV pausing?

**Figure 7:**
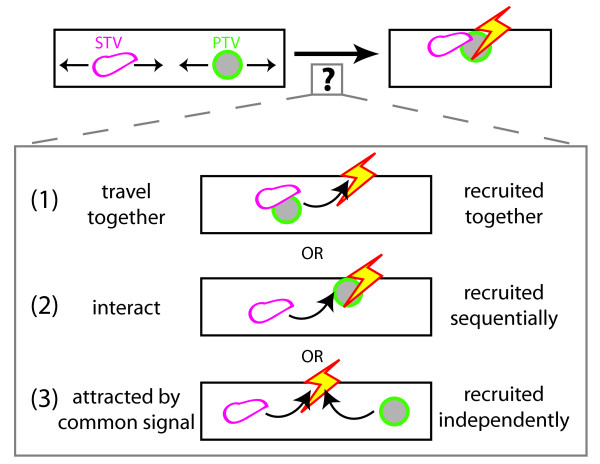
**Models for coordination of recruitment of STVs and PTVs to the same place at the same time**. STVs and PTVs could be simultaneously attracted to a given site by (1) attraction of STVs and PTVs that are co-transported; (2) sequential recruitment via a physical interaction between STVs and PTVs; and (3) simultaneous but independent recruitment in response to a common signal. Our data are most consistent with the third model.

Our data indicate that PTVs preferentially pause at sites where an STV is present. Likewise, STVs preferentially pause at sites where a PTV is present. There are at least three potential hypotheses that could account for this phenomenon (Figure 7). First, pausing of STVs and PTVs at the same sites could be a result of STVs and PTVs traveling together and consequently being recruited together. Second, it is possible that a paused vesicle can interact with and stabilize a moving vesicle. In this scenario, a local signal could cause one type of vesicle to pause at a specific site within the axon. That paused vesicle could then interact with other vesicles and cause them to pause at the same site. Third, STVs and PTVs could be independently attracted to a common signal within the axon. In this case, both STVs and PTVs would respond to that signal by preferentially pausing at the site of the signal, regardless of the presence of other vesicles. This, in turn, would increase the probability that either vesicle was present at the site of the signal and, therefore, the chance STVs and PTVs are simultaneously paused at the same site. Each hypothesis represents a viable mechanism through which both synaptic vesicle and active zone proteins could be recruited to the same site in the axon.

Our data are most consistent with the hypothesis that STVs and PTVs are recruited to the same sites at the same time as a consequence of responding to a common local signal. The first mechanism - simultaneous recruitment of STVs and PTVs that are transported together - cannot by itself explain coincident pausing since in many cases the STVs and PTVs that paused together paused sequentially rather than simultaneously (for example, Figure 3A,B(1 and 3)). To distinguish between the remaining two mechanisms, we reduced the density of PTVs within the axon, using a dominant negative construct corresponding to the SBD of syntabulin. If PTVs interact with STVs to influence STV pausing and recruitment to a given site, then decreasing PTV density should substantially alter STV pausing. However, in SBD-transfected axons, STVs displayed only small differences in pause duration and no change in the frequency of pausing when compared to STVs in control axons. Although differences in pause duration and velocity were statistically significant, the relatively subtle change in STV pausing suggests that PTVs exert only a small influence on STV pausing. This argues against the second hypothesis, in which STV and PTV pausing is exclusively controlled by an interaction between STVs and PTVs. However, based on the data, it is possible that STVs and PTVs respond to both local signals and one another.

It will be important in the future to identify the signals that recruit STVs and PTVs. It is not yet known which signals cause vesicles to pause. Potential signals include calcium, phosphorylation or small GTPase activity. Although it was previously shown that calcium could increase the duration of pausing, the probability of pausing was unchanged by reducing intracellular calcium [4], suggesting that calcium alone may not be responsible. Phosphorylation is known to regulate the activity of microtubule motor proteins and their association with microtubules or vesicles [24-28], raising the possibility that phosphorylation of motor proteins is the ultimate mechanism by which local signals cause STVs and PTVs to stop their transport. Small GTPases are well-known for their ability to control vesicle targeting [29-31], and recent work has shown that in *C. elegans*, arl-8, an Arf-like small G-protein, controls where along the axon synaptic proteins aggregate [32].

### Why don't all vesicles pause at any given site?

Although the majority of STVs and PTVs that encounter pause sites will pause at them, many vesicles ignore the pause sites and continue their movements. This is even true for stopping and recruitment that occurs in response to synaptogenic adhesion or axo-dendritic contact [4]. It is not clear why. One possibility is that there could be a limited number of 'docking sites' at each pause site. This seems unlikely since some vesicles pass by even while others can still pause. Alternatively, STVs and PTVs could exist in multiple states, at least one of which is unresponsive to local signals that cause pausing. PTVs and STVs display heterogeneity in apparent size, velocity, and pausing properties, which has been noted here and elsewhere [4,9,11,13,14,19,21]. These apparent differences may correlate with structural differences [16]. In general, smaller/dimmer vesicles appear more likely to move quickly and pass by pause sites without pausing. Perhaps these apparently smaller vesicles are unable to respond to the cues that induce pausing. Such a state could be a result of differences in their motors or in other proteins associated with the STVs or PTVs. The idea of STVs and PTVs as heterogeneous subpopulations will be an interesting topic for further exploration.

## Conclusions

For synapses to form, all of the components of presynaptic terminals must be delivered rapidly to the site of synapse formation. Despite its importance, we are only beginning to understand how this occurs. Here we have proposed a model for presynaptic terminal assembly in which trafficking of synaptic vesicle and active zone proteins is coordinated even prior to axo-dendritic contact. This coordination occurs through a combination of co-transport, perhaps in aggregates of vesicles tethered together [16], and co-pausing in response to local signals. This coordination then can facilitate presynaptic terminal assembly and contribute to the rapid recruitment of synaptic components that has been consistently observed. The same signals that induce co-pausing prior to transport may act downstream of synaptogenic adhesion to simultaneously attract STVs and PTVs to sites of synapse assembly.

## Materials and methods

All studies were conducted with an approved protocol from the Case Western Reserve University Institutional Animal Care and Use Committee, in compliance with the National Institutes of Health guidelines for the care and use of experimental animals.

### Neuronal cultures and transfection

Primary neuronal cultures were prepared from postnatal rat visual cortex essentially as described previously [4,5,15], except neurons were maintained in Neurobasal-A medium supplemented with glutamax and B27 (Invitrogen, Carlsbad, CA, USA). Neurons were transfected with Lipofectamine 2000 (Invitrogen) 24 to 48 h before live imaging. Excluding GFP-bassoon transfection, 1 μg of DNA construct was combined with 1 μg of Lipofectamine 2000 in 50 μl of Optimem (Invitrogen) for each 18-mm coverslip. Transfection of GFP-bassoon was conducted in the same manner except 2 μg of DNA were used to account for its large size. With double transfection, localization of each protein appeared similar when expressed alone. Also, the distribution and movement of STVs labeled with synaptophysin-mcherry, synaptophysin-mRFP, and synaptophysin-GFP all appeared similar to one another. GFP-bassoon (GFP-Bsn 95-3938), synaptophysin-mcherry, synaptophysin-mRFP, synaptophysin-GFP and syntaxin-SBD-GFP were generous gifts of Drs Thomas Dresbach (University of Heidelberg), Matthijs Verhage (Vrije Universiteit, Amsterdam), Jurgen Klingauf (University of Muenster), Jane Sullivan (University of Washington, Seattle) and Zu-Hang Sheng (National Institute of Neurological Disorders and Stroke, Bethesda). Each of these constructs has been shown previously to be functional and properly localized [15,19,20,22,33,34].

### Live imaging

Neurons were imaged at 7 to 8 DIV with a C1 Plus confocal system with a Nikon Eclipse Ti-E microscope using a 40× Nikon Plan Apo 0.95 numerical aperture objective. Lasers were 488 nm argon and 543 nm helium-neon. Detection filters were 515/30 nm bandpass for GFP and 590/50 nm bandpass for mcherry/mRFP. Images were collected every 10 s, with scan times no greater than 3.3 s. This imaging interval was selected as a compromise between having a high temporal resolution and minimizing the time the neurons were exposed to the laser to avoid any toxicity and photobleaching. A total of 45 images were collected for each time-lapse series. Imaging was conducted with constant perfusion with artificial cerebrospinal fluid (120 mM NaCl, 3 mM KCl, 2 mM CaCl_2_, 2 mM MgCl_2_, 30 mM D-glucose, 20 mM HEPES, and 0.2% sorbitol, pH 7.3). Artificial cerebrospinal fluid perfusion was performed at 25°C since STV transport and pausing are not significantly different at ambient and physiological temperatures. Axons were identified using morphological criteria, as described previously [4,15]. For dual-color imaging, channels were collected sequentially to eliminate bleed-through and neurons were imaged in which expression levels of both fusion proteins appeared comparable.

### Immunofluorescence

Neurons were fixed for 15 minutes in 4% paraformaldahyde in phosphate-buffered saline containing 4% sucrose, permeabilized for 5 minutes with 0.2% Triton X-100, and blocked with 10% horse serum. The primary antibody was piccolo (Synaptic Systems, Goettingen, Germany, and the secondary antibody was Alexa 633-conjugated goat anti-rabbit (Invitrogen). Coverslips were mounted in Fluoromount (Fisher Scientific, Pittsburgh, PA, USA) containing DABCO (1,4-diazabicyclo[2.2.2]octane) (Sigma, St Louis, MO, USA).

### Analysis and statistics

STV and PTV movements were tracked using ImageJ (NIH, Bethesda, MD, USA). To restrict the quantification of time-lapse movies to healthy neurons, axons were included in the analysis only if a least one vesicle moved within the field of view. Regions of axon with an intermediate STV or PTV density were imaged to allow us to track each vesicle reliably. STV and PTV movements were tracked independently while blind to the other channel. Movements were not analyzed if the axon moved significantly.

For quantification of movement and pausing, positional data were imported into Matlab and analyzed with custom-written programs (Mathworks, Natick, MA, USA). For pause analysis, a pause was defined as a period ≥10 s, during which the velocity of the vesicle went to 0 ± 0.1 μm/s. The cutoff zero velocity (0.1 μm/s) was chosen based on the average diameter of vesicles, which was approximately 1 μm. Vesicles that never moved were not included in the analysis. Therefore, vesicles which pause for very long durations might be underrepresented. Also, only vesicles that could be tracked for the entire imaging duration were included in the analysis. Vesicles that move at high velocities are more likely to leave the imaging area before the end of the movie. Therefore, these vesicles might also be underrepresented in the analysis.

Given the pixel density, scan speed and average vesicle size, a vesicle was typically imaged in approximately 10 to 50 ms, permitting reliable tracking of vesicles based on their apparent size, shape, and intensity, with relatively low influence of vesicle movements on these parameters. The fastest STV movements, at a maximum approaching 1 μm/s (comparable with the fastest velocities that have been reported for STVs [4,9,17]), resulted in movements of 10 μm during the imaging interval and were easily measured. Maximal velocities of PTVs are lower than maximal STV velocities [11,19] and were also easily recorded.

Data are presented cumulatively with 95% confidence intervals for binomially distributed data or as the mean ± standard error of the mean where appropriate. Confidence intervals were calculated based on a normal approximation, and data sets were considered significantly different if their 95% confidence intervals were non-overlapping. For data presented as means, significance was evaluated using the Wilcoxon rank sum test.

### Randomized model

In some cases, experimental data were compared to randomized simulations of vesicle movement. For each axon that was imaged, a model axon was generated with the same number of vesicles and axonal length as each experimental axon. In these models, the initial position of each vesicle was randomized. However, the number, timing and direction of movements, as well as the instantaneous velocity, and pause duration of each vesicle remained the same. The newly generated time-lapse series were then analyzed in the same manner as the experimental data. Each simulation was performed 100 times per axon to enhance statistical analysis.

## Abbreviations

DIV: days *in vitro*; GFP: green fluorescent protein; mRFP: monomeric red fluorescent protein; PTV: piccolo-bassoon transport vesicle; SBD: syntaxin binding domain; STV: synaptic vesicle protein transport vesicle.

## Competing interests

The authors declare that they have no competing interests.

## Authors' contributions

LB and SS performed experiments and analysis and wrote the paper. Both authors read and approved the final manuscript.

## Supplementary Material

Additional file 1 **Movie 1. Movie showing STV and PTV movements in the same axon**. The top three panels show bassoon, synaptophysin and the transmitted light image, respectively. The bottom panel corresponds to the overlay of all three. STVs and PTVs can be seen moving together and pausing at the same sites. The yellow boxes highlight areas in which STVs and PTVs clearly move together. The axon itself does not move significantly during the imaging. Times are indicated at the bottom left. Click here for file
